# Note on Cucumber Ointment

**Published:** 1853-10

**Authors:** 


*Note on Cucumber Ointment.*—Several years ago, (April, 1847,) Mr. Proctor published in this Journal a note on the preparation of Cucumber Ointment, since when it has gradually come more into use as an emollient application to irritated parts of the skin. It may not be improper to again call attention to the preparation and the mode of preparing it.

| | | | |
|---|---|---|---|
| Take of | Green Cucumbers, (suitable for table use,) | 7 | pounds, *av.* |
| " | Lard, (the purest and whitest,) . . . . | 24 | ounces, " |
| " | Veal suet, (selected,) . . . . . . . . . . | 15 | ounces, " |

The unpared cucumbers, after being washed, are reduced to a pulp by grating, and the juice expressed and strained. The suet is cut in small pieces, and heated over a salt water bath until the fat is fused out from the membranes; the lard is then added, and when liquefied is strained through muslin into a wide-mouthed earthen vessel capable of holding a gallon, and stirred until it commences to thicken, when one-third of the cucumber juice is added, and beaten with the ointment, by means of a wooden spatula, until its odor has been almost wholly extracted. The part that separates by standing, is decanted, and the other two thirds consecutively incorporated and decanted, in the same manner. The jar is then closely covered and placed in a water bath until the fatty matter entirely separates from the exhausted juice. The green albuminous coagulum, which floats on the surface, is then skimmed off, and the jar put aside in a cool place that the ointment may solidify. The crude ointment is then separated from the watery liquid on which it floats, melted, and strained; a part into a jar and closely sealed for keeping, the remainder into a mortar, and triturated with a little rosewater until it is very white and creamy, for present use. It is usual to keep this ointment in glass jars, covered with rosewater, to prevent access of the air.—*American Journal of Pharmacy*, from *Med. Examiner*.

